# Efficacy and Safety of Clearing Heat and Detoxifying Injection in the Treatment of Influenza: A Randomized, Double-Blinded, Placebo-Controlled Trial

**DOI:** 10.1155/2014/151235

**Published:** 2014-11-25

**Authors:** Yu Liu, Yuhong Huang, Baolin Wei, Xinqiao Liu, Yaqing Zhang, Xiaomin Huang, Yuping Tan, Zengtao Sun

**Affiliations:** ^1^The Second Affiliated Hospital of Tianjin University of Traditional Chinese Medicine, No. 816 Zhenli Road, Hebei District, Tianjin 300150, China; ^2^The First Affiliated Hospital of Tianjin University of Traditional Chinese Medicine, No. 314 Anshanxi Road, Naikai District, Tianjin 300193, China; ^3^Jiangsu Hospital of Integrated Traditional Chinese and Western Medicine, No. 100 Shizi Street, Hongshan Road, Nanjing 210028, China; ^4^The First Affiliated Hospital of Zhejiang University of Traditional Chinese Medicine, No. 54 Youdian Road, Shangyou District, Hangzhou 310006, China; ^5^Ruikang Hospital Affiliated to Guangxi University of Chinese Medicine, No. 10 Huadong Road, Nanning 530011, China

## Abstract

*Objective*. To evaluate the efficacy and safety of CHDI in the treatment of influenza infection. *Method*. A randomized double-blind, double dummy trial was conducted. Influenza patients with a positive influenza A rapid test diagnosis were randomized to receive CHDI or oseltamivir. Primary outcome was assessed by the median fever alleviation time and clearance time, and secondary outcome was total scores of influenza symptoms. *Results*. One hundred thirty-nine participants were screened and 34 had a RT-PCR laboratory confirmation of influenza virus infection. Fever alleviation time was 2.5 and 5 hours in CHDI and oseltamivir, respectively, and fever clearance time was 32.5 and 49 hours. The HR of fever alleviation and clearance time shows no significant difference between two groups. Total scores of influenza symptoms descended significantly in both groups after treatment and descended more in CHDI than oseltamivir on day 2. Similar to total symptoms severity score, fever severity score descend more significantly in CHDI than oseltamivir on day 2, and there were no differences on other symptoms. *Conclusions*. CHDI have a similar effect to oseltamivir in reducing the duration of influenza illness. CHDI was well tolerated, with no serious adverse events noted during the study period.

## 1. Introduction

The influenza virus is an orthomyxovirus and causes an acute respiratory tract disease. Influenza illness is characterized by abrupt onset of fever, headache, myalgia, sore throat, and nonproductive cough, whose complications often cause hospitalization and deaths in elderly and children, including bronchitis and pneumonia. Because annual influenza virus causes significant morbidity and mortality worldwide [1–3] and confers a considerable burden on health care systems [4–6], there is a need for effective and well-tolerated treatments that can reduce the impact of influenza on the individual and society.

**Figure 1:**
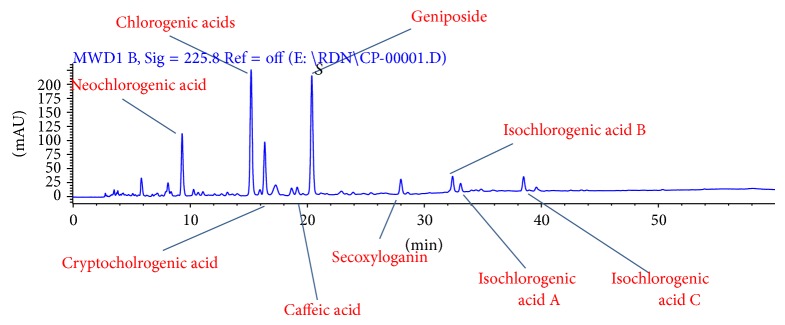
Liquid quantitative fingerprint of CHDI. CHDI, clearing heat and detoxifying injection. Nine ingredients in CHDI: (1) neochlorogenic acid, (2) chlorogenic acids, (3) cryptochlorogenic acid, (4) caffeic acid, (5) geniposide, (6) secoxyloganin, (7) isochlorogenic acid B, (8) isochlorogenic acid A, and (9) isochlorogenic acid C.

Traditional Chinese medicine (TCM) has been used in China for centuries to treat colds and influenza. Traditional Chinese medicine injection is a kind of new TCM preparations, which was mainly used for treatment of acute and severe disease. Clearing heat and detoxifying injection (CHDI), whose Chinese pinyin name is called Reduning injection, was approved by CFDA (China Food and Drug Administration) in May 2005 for the treatment of upper respiratory tract infection. To date, CHDI was prescribed to nearly 20 million patients in China, and the incidence of adverse reactions was nearly 0.4%, mainly skin itching. Nine ingredients of CHDI were listed in finger-print for quality control in Figure 1. CHDI is a key product of Jiangsu Kangyuan pharmaceutical co., LTD., which locates in Jiangsu province, east of China. Antiviral activity of extract from three plants that composes CHDI has been reported against herpes simplex virus, adenovirus, respiratory syncytial virus, parainfluenza virus, and influenza A viruses in many vivo and vitro studies [7–9]. Standardized CHDI has also been shown to inactivate or inhibit the proliferation of influenza virus FM, respiratory syncytial virus, and adenovirus in vivo study [10, 11]. Moreover, the mechanism research has reported that CHDI has a similar role in inhibiting neuraminidase (NA) as oseltamivir [12]. For influenza-like symptoms, the efficacy of CHDI was also demonstrated in small sample trials [13, 14].

We designed the current trial to test the hypothesis that CHDI, which is already on the market, would be well-tolerated and would reduce the duration of naturally acquired influenza illness. The study was conducted to determine safety and clinical efficacy of CHDI administered for 3 days in adults with microbiologically proven influenza.

## 2. Method

### 2.1. Study Design and Sites

A prospective, multicentre, double-blinded, double-dummy, randomized trial was conducted according to the revised Declaration of Helsinki and the approval of the ethical committee of the Second Affiliated Hospital of Tianjin University of Traditional Chinese Medicine in Sep. 2010. The subjects were enrolled from Jan. 2011 to Mar. 2011, when the epidemic of influenza in China was reported by Chinese Center for Disease Control and Prevention. The registration number is ChiCTR-TRC-10001082.

### 2.2. Patients

According to “The diagnosis and treatment guidelines for pandemic influenza” issued by the Ministry of Public Health of China, influenza was defined as one of the following conditions in epidemic period of influenza. (1) Generally characterized by acute high fever (axillary temperature 38°C), chills, headache, dizziness, ache all over, poisoning symptoms such as fatigue, sore throat, dry cough, and other respiratory symptoms, but catarrhal symptoms are often not obvious; (2) a few cases may have loss of appetite, abdominal pain, bloating, vomiting and diarrhea, and other gastrointestinal symptoms; (3) A few cases may also be complicated by sinusitis, otitis media, laryngitis, bronchitis, pneumonia, respiratory and circulatory failure, and even death; (4) chest X-ray examination of severe patients may show unilateral or bilateral pulmonary parenchymal disease, and a few may be associated with pleural effusions; (5) white blood cell in peripheral blood is not high or low, accompanied by relatively increased lymphocytes.

The inclusion criteria were (1) previously healthy adults aged 18 to 65; (2) onset of influenza symptoms within 48 hours; (3) axillary temperature that was 38.5°C or higher; (4) at least one or more respiratory symptoms (sore throat, cough, and nasal congestion); (5) at least one or more general symptoms (headache, fatigue, and myalgia); (6) a positive throat rapid test for influenza A performed by the practitioner (Clear view Exact Influenza A&B).

The exclusion criteria were (1) receiving influenza vaccination 12 months prior to the beginning of the study; (2) routine blood WBC that was greater than the upper limit of normal value; (3) having chronic respiratory diseases or pneumonia; (4) having clinically significant chronic illness or human immunodeficiency virus disease; (5) receiving systemic steroids or other immunosuppressants 3 months prior to the beginning of the study; (6) women who had a positive urine pregnancy test before drug administration.

Prior to inclusion, patients gave informed written consent.

### 2.3. Treatment

Qualified patients were randomly allocated to treatment or control group. In treatment group, patients received simulation agent of oseltamivir plus CHDI. In control group, patients were given oseltamivir plus simulation agent of CHDI. Both oseltamivir and its simulation were administered 75 mg orally twice a day for 5 days. CHDI was 20 mL added in normal saline 250 mL, and simulation of CHDI was saline 250 mL. Both were intravenously administered by research nurses, once a day for 3 days, and the infusion lasted nearly 90 minutes. On days 1, 2, and 3, patients received CHDI or its matching placebo at hospital. On days 4 and 5, they only took oseltamivir or its simulation agent at home. On day 6 or day 7, they got back to hospital for follow-up examination.

Seven hours after the first time use of study medication, patients would be instructed to take paracetamol if their axillary temperature was still above 39°C. The use of paracetamol and any other medications was recorded in patient dairy card. Compliance was assessed by checking patient records of the date and time of each dose and verified by counting capsule returns for each patient.

### 2.4. Clinical Monitoring

Researchers accessed and recorded the severity score of 8 influenza symptoms of patients at baseline (before treatment on day 1) and once daily before treatment on day 2 and day 3, when patients came to hospital for medication infusion. A 4-level score was applied in accessing the severity of every symptom: fever (0, <37.2°C; 3, 37.3~37.9°C; 6, 38.5~38.9°C; 9, above 39°C); being afraid of the cold and myalgia (0, absent; 2, mild; 4, moderate; 6, severe); cough, nasal obstruction, sore throat, fatigue, and headache (0, absent; 1, mild; 2, moderate; 3, severe).

Axillary temperature was taken by patients with a digital thermometer and recorded in patient diary card during the study, 8 times on day 1, 6 times on day 2, and twice on the other days during dosing period.

### 2.5. Randomization and Blinding

Patients were randomized according to a predefined computer-generated randomization list with the balanced 1 : 1 randomization using a block size of four. A research pharmacist at Nanjing Medical University received the study medication from the producer of CHDI, Jiangsu Kangyuan pharmaceutical co., LTD., prepared the study medication according to the randomization schedule and then shipped study medication to the clinical site, which distributed the numbered container of study medication to research nurses sequentially, when eligible participants were enrolled.

Because the colour of CHDI is light yellow, the brown infusion tube was applied in infusion operation process to avoid breaking the blinding. Research nurses, who operated the infusion, did not take part in the evaluation process in the trial. Besides, simulation agent and oseltamivir had an identical appearance and taste. Simulation agent of oseltamivir was made by Jiangsu Kangyuan pharmaceutical co. LTD, which did the blinding test in accordance with the drug quality standard approved by CFDA and issued the test report.

### 2.6. Laboratory Method

Posterior pharyngeal throat swabs for isolation of influenza virus were taken at baseline. Swabs were taken from enrolled patients' throat, placed into 3 mL of viral transport medium, and transported at 4°C by special courier to the National Influenza Centre (NIC) of China. Upon arrival, the swab samples were eluted into 2 mL of transport medium, processed for real-time reverse transcription- (RT-) PCR analyses, and inoculated onto MDCK cells for virus isolation and subsequent subtyping using a standard hemagglutination inhibition assay. For RT-PCR analyses, RNA extraction from 200 *μ*L of specimen was performed using the QIAmp viral RNA mini kit (Qiagen) with RNA elution into a final volume of 60 *μ*L. All real-time RT-PCR assays were performed in a final volume of 15 *μ*L with 5 *μ*L RNA, 0.4 *μ*M of each primer, 0.2 *μ*M probe, and 0.8 *μ*L enzyme mix (SuperScript III platinum one-step quantitative RT-PCR system, Invitrogen). Type A influenza virus RNA was detected by a real-time RT-PCR targeting the conserved matrix gene using GRAM/7Fw (5′-CTTCTAACCGAGGTCGAAACGTA-3′) and GRAM/161Rv (5′-GGTGACAGGATTG GTCTTGTCTTTA-3′) primers and GRAM probe/52/+ (5′[Fam]-TCAGGCC CCTCAAAGCCGAG-[BHQ-1]3′) probe.

#### 2.6.1. Case Definition

For the primary outcome analysis, laboratory influenza infection was defined as isolation of influenza virus from throat secretions.

### 2.7. Efficacy End Points

The primary outcome was evaluated by assessing the median fever alleviation time and clearance time, and the secondary outcome was total scores of all influenza symptoms on each visit time. The fever alleviation time was defined as time from baseline until the first time axillary temperature descended more than 0.5°C. The fever clearance time was defined as time from baseline until the first time axillary temperature fell below 37.4°C and remained below 37.4°C for at least a further 24 hours.

#### 2.7.1. Case Definition

For the primary outcome analysis, laboratory influenza infection was defined as isolation of influenza virus from throat secretions.

### 2.8. Statistical Analysis

The primary outcomes were carried out for patients who received at least 1 dose of study drug and had laboratory confirmed influenza infection. The secondary outcome analysis was performed for all subjects who received study drug irrespective of laboratory evidence of infection. Patients who received at least one time drug were included in the safety assessment.

The fever alleviation and clearance time were expressed as P50, using univariate COX regression model comprising time-censored data to analyze the differences between two groups. Variables assumed to be continuous were expressed as mean values with 95% confidence intervals constructed using Student's* t*-distribution method. The standard deviation and total range were used as indices of distribution. Intergroup analyses were carried out using two-tailed tests with a significance level of 5%. All participants who received at least one time drug were included in the safety assessment.

SAS (version 6.0) software (Statistical Analysis System, SAS Institute, Cary, NC, USA) was used for all the statistical analyses.

## 3. Results

### 3.1. Participant Enrollment Flow

**Figure 2:**
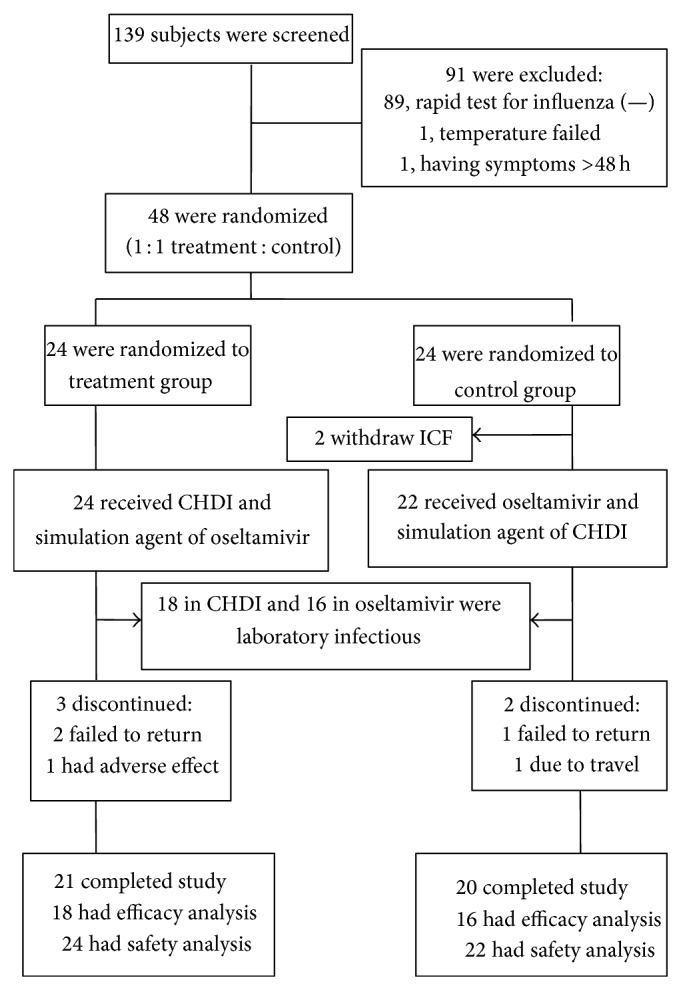
Participants flow.

139 participants were screened from 5 centers. Among them, influenza rapid test of 48 (34.5%) participants was positive, who were randomized to CHDI (*n* = 24) and oseltamivir (*n* = 24). 34 participants (18 in CHDI and 16 in oseltamivir) had laboratory confirmation of influenza virus infection on the baseline specimen. 41 participants completed the study (21 in CHDI and 20 in oseltamivir) (Figure 2).

### 3.2. Baseline Data

No significant differences of demographic characteristics and symptoms scores were observed between CHDI and oseltamivir groups. None of the participants used drug before enrollment. Axillary temperature of influenza-infected participants in CHDI and oseltamivir is 38.66 ± 0.22°C and 38.78 ± 0.30°C, respectively, duration of illness prior to enrollment is 15.50 ± 14.00 and 19.00 ± 13.00 hours, respectively, and total symptoms severity score is 20.29 ± 4.71 and 20.35 ± 4.83, respectively (Table 1).

### 3.3. Primary Outcome

Among influenza-infected participants, the fever alleviation time was 2.5 and 5 hours (P50) in CHDI and oseltamivir groups, respectively, and the median fever clearance time was 32.5 and 49 hours (P50), respectively. The HR (hazard ratio) of fever alleviation time was 0.52 and 95%CI was from 0.25 to 1.08. The HR of fever clearance time was 0.69 and 95%CI was from 0.34 to 1.43. HR of both groups are less than one, but there is no statistical differences between two groups (*P* > 0.05). Compared with oseltamivir treatment group, CHDI treatment group showed a trend of decline in fever alleviation and clearance time (Table 2).

Because it is likely that an antiviral drug to treat influenza would be used in the absence of laboratory microbiologic diagnosis, we also performed an analysis of the effect on treatment on all participants who received medication regardless of microbiologic results. Similarly in all participants, individuals receiving CHDI treatment showed a trend of rapid returning to normal body temperature (Table 2).

### 3.4. Secondary Outcome

Compared with before treatment (baseline), both CHDI and oseltamivir groups reduced the total symptoms severity score significantly from day 2 to day 6 (*P* < 0.0001). Comparing between groups, total symptoms severity score descended significantly in CHDI group more than in oseltamivir group on day 2 (*P* < 0.05). The decline of CHDI group on day 2 was 9.90 ± 5.3, more than 8.70 ± 4.58 in oseltamivir group. No significant difference was shown on day 3 and day 6 between the two groups (Table 3).

**Figure 3:**
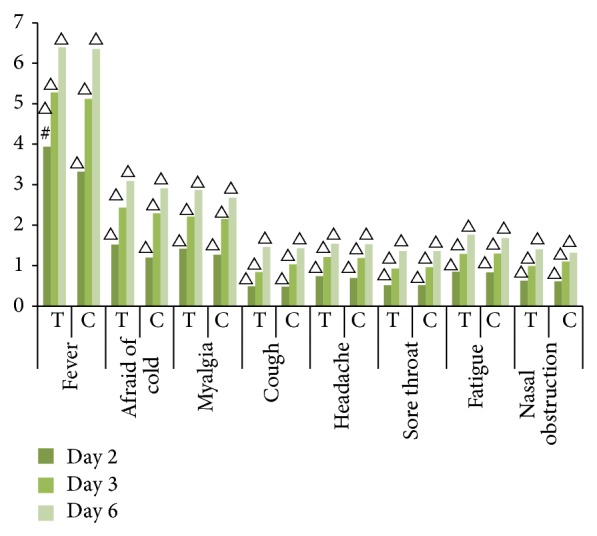
Decline of single symptom severity score. T, treatment group (CHDI); C, control group (oseltamivir); ^#^between two groups, comparing with control group, fever severity score of treatment group descended significantly on day 2 (*P* < 0.005). ^△^within the group itself, comparing with prior treatment score, posttreatment score descended significantly on day 2, day 3, or day 6 (*P* < 0.0001).

Alleviation of single symptom was similar to the downward trend of total symptoms severity score. CHDI and oseltamivir groups reduced severity score of every symptom significantly from day 2 to day 6 (*P* < 0.0001) when compared with before treatment, but no significant difference between two groups, except for fever on day 2, 3.94 ± 1.99 in CHDI and 3.32 ± 1.90 in oseltamivir (<0.05), was observed (Figure 3).

### 3.5. Adverse Event

Clearing heat and detoxifying injectionand oseltamivir were well-tolerated in all participants. In CHDI group, there was one transfusion reaction, shown as light headache, sweating, nausea, and low blood pressure of 84/54 mmHg and this participant becomes normal after stopping infusion. Blood leukocytes of two participants (1 from CHDI group and 1 from oseltamivir group) descended slightly after treatment. In the study, no serious drug-related adverse events occurred and there was no use of rescue medication.

## 4. Discussion

This is the first multicenter, randomized controlled, double-blinded study in which TCM injection was administered to influenza patients who were etiology diagnosed, using oseltamivir as a positive control drug, which is now still accepted as effective drug for the treatment of influenza [15].

### 4.1. Summary of Main Findings

The results of this study indicate that infusion CHDI may be an effective treatment for influenza in adults as oseltamivir. Both of CHDI and oseltamivir can shorten the duration of influenza. The fever alleviation time of CHDI is 2.5 h (P50) and that of oseltamivir is 5 h (P50). The fever clearance time of CHDI and oseltamivir is 32.5 h (P50) and 49 h (P50), respectively. HR of fever alleviation and resolution time is 0.52 and 0.69. Though no statistical difference of HR has been shown, the study gave the research practitioner confidence to prescribe CHDI to influenza patient.

Both CHDI treatment and oseltamivir treatment resulted in alleviation of all influenza symptoms, including being afraid of cold, myalgia, cough, headache, score throat, fatigue, and nasal obstruction. For relieving fever, CHDI may be better than oseltamivir within 24 h after the first treatment, while similar benefits that result from oseltamivir were confirmed by a parallel trial, which was conducted in Canada and Europe during influenza season [16].

### 4.2. Relationship between Our Study and the Existing Literature

Comparisons of our study results with those of other antiviral approaches to influenza are complicated by differences between trials in the specific outcome measurements and the variable nature of influenza illness each year. Besides, to our knowledge, no oseltamivir-controlled, double-blinded study has been done with TCM remedies for influenza viruses. However, CHDI for reliving symptoms in this study, such as fever and cough, was comparable to those described in treatment of respiratory tract infection by CHDI in China [17, 18].

The documented infusion reactions of CHID reported in other articles, such as light headache, sweating, and nausea [19], were also observed in one subject of our study. Infusion reaction disappeared after drug was suspended, without any treatment, while no research has found that CHDI has the risk of reducing blood leukocytes. Therefore, it can be speculated that slight change of blood leukocytes of 1 subject in CHDI group was not caused by CHDI.

### 4.3. Weaknesses of the Study

Several potential limitations of this study should be considered. Screening patients who had rapid test confirmation of influenza virus infection is relatively low compared with other studies [20, 21], and virus replication using RT-PCR in the upper respiratory tract has not been assessed during treatment. It is worth mentioning that sore throat in CHDI group reduced as well as oseltamivir 75 mg treatment, which has been proved to be effective in reducing influenza virus in related studies [22].

Lost time from work or school and reductions in performance represent important effects of influenza on healthy adults [23, 24], while our study was not specifically designed to evaluate the economic benefit in reducing lost time from work. However, patients in CHDI group were accompanied by the quick return to normal activities as those in oseltamivir group. To certain extent, it suggested that CHDI might reduce economic loss due to influenza. Therefore, we hope to observe economic benefit of CHDI in future study.

### 4.4. Implications from the Study

Though vaccination is effective for prophylaxis and in reducing the impact of influenza, it is used rarely in low-income settings. Besides, some elderly individuals and immunocompromised people do not respond optimally to the vaccine, and the vaccine may not always include the strain of virus circulating within a given community [25]. Amantadine and rimantadine interfere with the replication cycle of type A influenza viruses, but they have no activity against influenza B viruses and are associated with the emergence of resistant viruses in treated individuals [26]. In addition, both drugs can cause central nervous system and gastrointestinal adverse effects, which may be more common in older individuals [27]. Zanamivir and oseltamivir belong to a new class of antiviral agents known as neuraminidase inhibitors. Though zanamivir is effective in the treatment of influenza [28–30], it must be administered topically (i.e., by inhalation or intranasally) or parenterally [31] to be effective. Oseltamivir is the oral prodrug which has clinical and virological benefits in patients with influenza when it is administered within 48 hours of onset of symptoms [20, 32], which also produces gastrointestinal adverse effects [33]. And recent studies show that oseltamivir is associated with rare but severe adverse effects of mental system in pediatrics [34].

In contrast to the above antiviral drugs, CHDI preliminarily shows a broad spectrum of antiviral effect in vitro and can be administered to the whole population, including children, suffering from pneumonia and treated with CHDI and other drugs in China [35, 36]. CHDI offers an efficient, safe supplement to the present armamentarium of medications for the prophylaxis and treatment of influenza. It should be stressed that our study involved only adult influenza patients who were otherwise healthy and did not include any high risk patients. Further studies are required to confirm these results in other patient groups and to assess virological efficacy.

## Figures and Tables

**Table 1:** Demographic characteristics of the participants and clinical characteristics of influenza-infected participants.

Characteristic	Study groups	
	CHDI, 30 mL (*n* = 24)	Oseltamivir, 75 mL (*n* = 22)
Age, mean (SD), y	37.9 (13.9)	38.4 (14.5)
Men, number (%)	12 (50.00)	7 (31.81)
Drug used before enrollment, number (%)	0 (0)	0 (0)
Merge other disease, number (%)	0 (0)	0 (0)
Infected, number (%)	18 (75)	16 (72)
Influenza A^§^	14	13
Influenza B	4	3

	Individuals infected with influenza	
	(*n* = 18)	(*n* = 16)

Duration of illness before study, mean (SD), h	15.50 (14.00)	19.00 (13.00)
Axillary temperature, mean (SD), °C	38.66 (0.22)	38.78 (0.30)
Total symptoms severity score at enrollment, mean (SD)	20.29 (4.71)	20.35 (4.83)

CHDI: clearing heat and detoxifying injection.

^§^Influenza A virus infections were predominantly SWL; 2 infections in oseltamivir group were due to H3N2 virus.

**Table 2:** Duration of fever symptom in CHDI and Oseltamivir groups.

	Influenza-infected participants		All treated participants	
	CHDI (*n* = 18)	Oseltamivir (*n* = 16)	CHDI (*n* = 24)	Oseltamivir (*n* = 22)
Fever alleviation time, *P*_50_ (*P*_25_~*P*_75_), h^*※*^	2.5 (1.5~3)	5 (3.5~10)	2 (1~3)	6 (3~8)
HR, (95% CI)/*P*	0.52 (0.25, 1.08)/0.07^#^		0.47 (0.36, 0.61)/0.001^#^	
Fever clearance time, *P*_50_ (*P*_25_~*P*_75_), h	32.5 (17.5~52)	49 (32.2~81)	29 (18~46)	49 (33.5~69)
HR, (95% CI)/*P*	0.69 (0.34, 1.43)/0.32^#^		0.64 (0.49, 0.83)/0.001^#^	

^*※*^ *P*_50_ indicates the median time. *P*_25_ and *P*_75_ indicate lower quartile and upper quartile. ^#^HR < 1, but *P* > 0.05 indicates that fever alleviation and clearance time are less in CHDI than in Oseltamivir, but no statistical differences were shown.

**Table 3:** Decline of total symptoms severity scores in the group itself and between two groups.

Time	group	*n*	PO − PR $\bar{X}\pm s$ (95% CI)	In the group itself		T − C $\bar{X}\pm s$ (95% CI)	Between groups	
				*t-*test	*P-*value		*t-*test	*P* value
Day 2	T	24	9.90 ± 5.30 (8.89, 10.91)	19.40	<0.0001	1.20 ± 0.67 (−0.11, 2.52)	4.18	**0.042** ^ #^
	C	22	8.70 ± 4.58 (7.84, 9.55)	20.09	<0.0001			

Day 3	T	24	14.84 ± 5.21 (13.85, 15.84)	29.62	<0.0001	0.09 ± 0.67 (−1.24, 1.42)	0.02	0.888
	C	22	14.75 ± 4.79 (13.85, 15.65)	32.57	<0.0001			

Day 6	T	24	19.41 ± 4.48 (18.55, 20.26)	44.99	<0.0001	0.64 ± 0.61 (−0.56, 1.84)	1.21	0.272
	C	22	18.77 ± 4.57 (17.91, 19.62)	43.43	<0.0001			

T: treatment group (CHDI); C: control group (oseltamivir); PR − PO, posttreatment score minus prior treatment score; $\bar{X}\pm s$. Mean ± SD, T − C: treatment group score minus control group score, and ^#^significant difference between groups.

## References

[1] Nichol K. L., Lind A., Margolis K. L., Murdoch M., McFadden R., Hauge M., Magnan S., Drake M. (1995). The effectiveness of vaccination against influenza in healthy, working adults. *The New England Journal of Medicine*.

[2] Harper S. A., Bradley J. S., Englund J. A., File T. M., Gravenstein S., Hayclen F. G., McGeer A. J., Neuzil K. M., Pavia A. T., Tapper M. L., Uyeki T. M., Zimmerman R. K. (2009). Seasonal influenza in adults and children-diagnosis, treatment, chemoprophylaxis, and institutional outbreak management: clinical practice guidelines of the infectious diseases society of America. *Clinical Infectious Diseases*.

[3] Thompson W. W., Shay D. K., Weintraub E. (2003). Mortality associated with influenza and respiratory syncytial virus in the United States. *The Journal of the American Medical Association*.

[4] Schoenbaum S. C. (1987). Economic impact of influenza. The individual's perspective. *The American Journal of Medicine*.

[5] Monto A. S. (1999). Individual and community impact of influenza. *PharmacoEconomics*.

[6] Neuzil K. M., Mellen B. G., Wright P. F., Mitchel E. F., Griffin M. R. (2000). The effect of influenza on hospitalizations, outpatient visits, and courses of antibiotics in children. *The New England Journal of Medicine*.

[7] Wang Y. Z., Cui X. L., Gao Y. J., Guo S. S., Wang X. K., Huang Y., Zhao Y., Gong W. F. (2006). Anti-virus test of gardenia extract. *China Journal of Chinese Materia Medica*.

[8] Zhang J. F., Zheng H. Y., Jian T. (2005). Inhibition mechanism of artemisinin on herpes simplex virus. *Jiangsu Pharmaceutical and Clinical Research*.

[9] Li Y. M., Li L., Bai C., Li D., Wang T. Z. (2001). Effect of extract from honey suckle flower on anti- adenovirus. *West China Journal of pharmaceutical Sciences*.

[10] Feng Y. Z., Zhou F., Huang M., Yao W. (2007). Inhibition effect of Reduning injection on influenza virus-FMI. *Chinese Journal of New Drugs and Clinical Remedies*.

[11] Feng Y. Z., Zhou F., Huang M., Yao K. (2007). Vitro inhibition effect of Reduning injection on adenovirus-3. *Chinese Journal of New Drugs and Clinical Remedies*.

[12] Sun L., Liu A. L., Wang Z. Z., Bi Y. A., Du G. H., Xiao W. (2014). Inhibition effect of Reduning injection and its components on influenza virus neuraminidase. *Modern Medicine and Clinical*.

[13] Huang X. M., Liu Y. J., He Y. Z., Chen F. F., Wang Y. K. (2006). Clinical observation of Reduning injection in treatment of patients with acute upper respiratory tract infections. *Chinese Journal of Clinical Pharmacology and Therapeutics*.

[14] Ying S. Y. (2006). Clinical observation of Reduning injection in treatment of children acute upper respiratory tract infection with fever. *China Medical Herald*.

[15] Ebell M. H., Call M., Shinholser J. (2013). Effectiveness of oseltamivir in adults: a meta-analysis of published and unpublished clinical trials. *Family Practice*.

[16] Aoki F., Osterhaus A., Rimmelzwaan G., Kinnersley N., Ward P. Oral GS4104 succesfully reduces duration and severity of naturally acquired influenza.

[17] Qian Y. Q. (2013). Verifing the efficacy of Reduning injection in the treatment 35 cases of acute respiratory infections. *Guide of China Medicine*.

[18] Cui B. Y., Gao K. W. (2013). Reduning injection in the treatment of pediatric respiratory tract infection in 120 cases. *Chinese Journal of Integrated Traditional and Western Medicine*.

[19] Tian W. Y. (2012). Adverse reactions of 93 cases reduning injection. *Guiding Journal of Traditional Chinese Medicine and Pharmacy*.

[20] Treanor J. J., Hayden F. G., Vrooman P. S. (2000). Efficacy and safety of the oral neuraminidase inhibitor oseltamivir in treating acute influenza: a randomized controlled trial. *The Journal of the American Medical Association*.

[21] Fry A. M., Goswami D., Nahar K., Sharmin A. T., Rahman M., Gubareva L., Azim T., Bresee J., Luby S. P., Brooks W. A. (2014). Efficacy of oseltamivir treatment started within 5 days of symptom onset to reduce influenza illness duration and virus shedding in an urban setting in Bangladesh: a randomised placebo-controlled trial. *The Lancet Infectious Diseases*.

[22] South East Asia Infectious Disease Clinical Research Network (2013). Effect of double dose oseltamivir on clinical and virological outcomes in children and adults admitted to hospital with severe influenza: double blind randomised controlled trial. *The British Medical Journal*.

[23] Smith A. P., Thomas M., Brockman P., Kent J., Nicholson K. G. (1993). Effect of influenza B virus infection on human performance. *British Medical Journal*.

[24] Keech M., Scott A. J., Ryan P. J. J. (1998). The impact of influenza and influenza-like illness on productivity and healthcare resource utilization in a working population. *Occupational Medicine*.

[25] Centers for Disease Control and Prevention (1998). Prevention and control of influenza: recommendations of the Advisory Committee on Immunization Practices (ACIP). *MMWR—Recommendations and Reports*.

[26] Hayden F. G., Belshe R. B., Clover R. D., Hay A. J., Oakes M. G., Soo W. (1989). Emergence and apparent transmission of rimantadine-resistant influenza A virus in families. *The New England Journal of Medicine*.

[27] Hayden F. G., Gwaltney J. M., van De Castle R. L., Adams K. F., Giordani B. (1981). Comparative toxicity of amantadine hydrochloride and rimantadine hydrochloride in healthy adults. *Antimicrobial Agents and Chemotherapy*.

[28] Hayden F. G., Osterhaus A. D., Treanor J. J., Fleming D. M., Aoki F. Y., Nicholson K. G., Bohnen A. M., Hirst H. M., Keene O., Wightman K. (1997). Efficacy and safety of the neuraminidase inhibitor zanamivir in the treatment of influenzavirus infections. *The New England Journal of Medicine*.

[29] Campion K., Silagy C. A., Keene O., Cooper C., Bolton P., Watts R., Lincoln P., Liaw T., Narayan K., Delooze F. (1998). Randomised trial of efficacy and safety of inhaled zanamivir in treatment of influenza A and B virus infections. *The Lancet*.

[30] Monto A. S., Fleming D. M., Henry D., De Groot R., Makela M., Klein T., Elliott M., Keene O. N., Man C. Y. (1999). Efficacy and safety of the neuraminidase inhibitor zanamivir in the treatment of influenza A and B virus infections. *The Journal of Infectious Diseases*.

[31] Calfee D. P., Peng A. W., Cass L. M. R. Protective efficacy of intravenous zanamivir in experimental human influenza.

[32] Nicholson K. G., Aoki F. Y., Osterhaus A. D. M. E., Trottier S., Carewicz O., Mercier C. H., Rode A., Kinnersley N., Ward P. (2000). Efficacy and safety of oseltamivir in treatment of acute influenza: a randomised controlled trial. *The Lancet*.

[33] Strong M., Burrows J., Stedman E., Redgrave P. (2010). Adverse drug effects following oseltamivir mass treatment and prophylaxis in a school outbreak of 2009 pandemic influenza A(H1N1) in June 2009, Sheffield, United Kingdom. *Eurosurveillance*.

[34] Wallensten A., Oliver I., Lewis D., Harrison S. (2009). Compliance and side effects of prophylactic oseltamivir treatment in a school in South West England. *Euro Surveillance*.

[35] Deng H. M. (2008). Efficacy of Reduning injection in the treatment of children with acute bronchopneumonia. *International Medicine & Health Guidance News*.

[36] Xiang J. F., Chen J., Dong Q., Wang Y. Z. (2011). Reduning injection in the treatment of children with viral pneumonia in 46 cases. *Zhejiang Journal of Traditional Chinese Medicine*.

